# Noun Sequence Statistics Affect Serial Recall and Order Recognition Memory

**DOI:** 10.1162/opmi_a_00092

**Published:** 2023-08-11

**Authors:** Steven C. Schwering, Maryellen C. MacDonald

**Affiliations:** Department of Psychology, University of Wisconsin–Madison, Madison, WI, USA

**Keywords:** working memory, short-term memory, language

## Abstract

Most theories of verbal working memory recognize that language comprehension and production processes play a role in word memory for familiar sequences, but not for novel lists of nouns. Some language emergent theories propose that language processes can support verbal working memory even for novel sequences. Through corpus analyses, we identify sequences of two nouns that resemble patterns in natural language, even though the sequences are novel. We present 2 experiments demonstrating better recall in college students for these novel sequences over the same words in reverse order. In a third experiment, we demonstrate better recognition of the order of these sequences over a longer time scale. These results suggest verbal working memory and recognition of order over a delay are influenced by language knowledge processes, even for novel memoranda that approximate noun lists typically employed in memory experiments.

## INTRODUCTION

Some memories persist for years while others fade in seconds. This striking dissociation is commonly explained via two distinct memory stores: long-term memory (LTM) and a dedicated temporary store (e.g., Baddeley & Hitch, 1974). Alternative proceduralist and emergent approaches have instead suggested that cognitive processes generate and maintain temporary memories, obviating the need for a separate temporary store (Cowan, 1995; Crowder, 1993). For example, language emergent accounts posit that encoding, maintenance, and recall in verbal working memory (VWM) tasks is supported by language comprehension and production processes (MacDonald, 2016; Majerus, 2013; Schwering & MacDonald, 2020). These two-system and emergent alternatives offer radically different views of memory and its interaction with other cognitive systems, so that evidence favoring one or the other may have important implications not only for theories of memory but also for theories of cognition more generally.

Emergent VWM approaches gained traction via results linking temporary memory to linguistic LTM. Neurally, brain areas supporting phonology and semantics also support VWM (Acheson et al., 2011; Buchsbaum & D’Esposito, 2019). Behaviorally, word properties learned from language experience and encoded in linguistic LTM support VWM performance, such as effects of phonological regularity on recall (Gathercole, 1995; Gupta & Tisdale, 2009; Page & Norris, 2009). These findings show that language processes and linguistic LTM underlie VWM performance (Majerus, 2013; Schwering & MacDonald, 2020), a significant departure from claims that VWM requires a separate temporary store (Baddeley & Hitch, 1974). Consequences of this shift are substantial for understanding individual differences; several studies suggest that tasks that were purported to measure VWM instead assess language experience (e.g., Klem et al., 2015).

However, there are several potential objections to these emergent accounts. Some researchers assume that the word lists employed in serial recall tasks, typically a series of nouns, “lack any sequential redundancy or meaningful structure” (Allen & Baddeley, 2009, p. 65), meaning that language processes alone cannot maintain memory for these unfamiliar lists in VWM tasks (Allen & Baddeley, 2009; Norris, 2017). They suggest that a dedicated temporary store is needed “to create novel structured representations that cannot yet be in LTM” (Norris, 2017, p. 1003). In other words, a separate temporary store is still needed for novel lists, which cannot be supported by linguistic LTM, on this view.

This account places VWM research in a curious state of theorizing. Whereas two-system approaches once distinguished LTM and VWM, researchers now posit two systems within VWM: LTM-guided maintenance for familiar words and orders, and a dedicated system for maintaining words and orders that are unfamiliar. Establishing the nature of “familiar” and “novel” sequences and the boundaries between and interactions among these two hypothesized working memory systems is a critical test of memory theories.

Two sets of psycholinguistic findings are relevant to these issues. The first concerns novelty and linguistic LTM. Sprouse et al. (2018) found that the novelty of word strings varies on a continuum, without a clear distinction between novel and familiar word strings. Relatedly, language comprehension becomes easier with word and word sequence acceptability (Hofmeister et al., 2013), reflecting improved encoding and interpretation with sequences that are judged more similar to prior experience with language. If there are separate systems maintaining memory for novel and familiar strings, it is unclear how the continuum of novelty would be partitioned into the two systems. In contrast, if the same language processing system handles both novel and familiar word strings, memory for novel and familiar sequences in VWM tasks may be governed by the same language systems.

The second set of findings addresses the relationship between individual words and their surrounding grammatical context. Specifically, word identity and its statistical patterns of word order are not independent: words are typically found in certain contexts—grammatical roles, sentence types, co-occurrences, and so on. These lexico-syntactic patterns, gleaned from experience and stored in LTM, strongly influence language processing and enable generalization to previously unencountered phrases and sentences (MacDonald, 1994). Relatedly, there are neighborhood effects across phonological patterns, semantics, and sentence structures; these statistics integrate word and word order in LTM, because efficient language use depends upon integrating word identity with context. Therefore, if language processes and linguistic LTM support VWM, word identity and context should interact to inform VWM, even when context is novel.

These word-context interactions are not merely a function of associations between words, which have been extensively shown to influence performance in VWM in the form of contextual diversity (Hulme et al., 2003; Stuart & Hulme, 2000), word-word associations (Majerus et al., 2012; Saito et al., 2020), or chunking (Jones & Macken, 2018). Rather, language users track functional relations between words in linguistic structures. For example, people track not just the probability of the exact noun sequence *recruitment officer*, but also understand that *recruitment* describes a particular kind of *officer*, given that noun-noun sequences often represent noun compounds in English. Importantly, these relationships are a function of both word properties and context, such that familiar words can aid processing of novel contexts and vice versa. That is, language users can extend and generalize the statistical or syntactic regularities they have learned to novel contexts (Wonnacott et al., 2008).

Together, this work on the importance of word-context statistics in linguistic VWM for comprehension and learning suggests that words and contexts should also support one another in VWM tasks (Schwering & MacDonald, 2020). The sentence superiority effect is generally consistent with this perspective. Participants recall arguably novel, sentence-like lists (Allen et al., 2018; Baddeley et al., 2009; Jones & Farrell, 2018), and grammatical regularities such as novel adjective-noun pairs (Perham et al., 2009; Schweppe et al., 2022), better than scrambled lists (Schwering & MacDonald, 2020). These results are consistent with involvement of linguistic LTM in sentence superiority effects.

However, an alternative interpretation of the sentence superiority effect again posits limited engagement of language processes. Jones and Farrell (2018) suggested that sentence superiority effects can emerge from LTM of part-of-speech sequences, like adjective, noun, and verb. This approach captures LTM of part-of-speech associations or chunks in VWM, but it ignores many of the additional constraints beyond part-of-speech that affect language use. It also has limited application to memory for noun lists, which are often employed in many VWM experiments. Two separate systems are again required to process memory for familiar and novel sequences.

In this study, we tested whether memory for words and their orders interact in ways consistent with processing of natural language. This approach emphasizes the importance of functional relationships among words and context. We created novel noun sequences in memory lists that were either consistent or inconsistent with statistical patterns of English. Employing only nouns in the critical manipulation removes the part-of-speech explanation for any differences between conditions and aligns our work with the noun lists used in many VWM studies (e.g., Allen & Baddeley, 2009).

**Figure 1.**
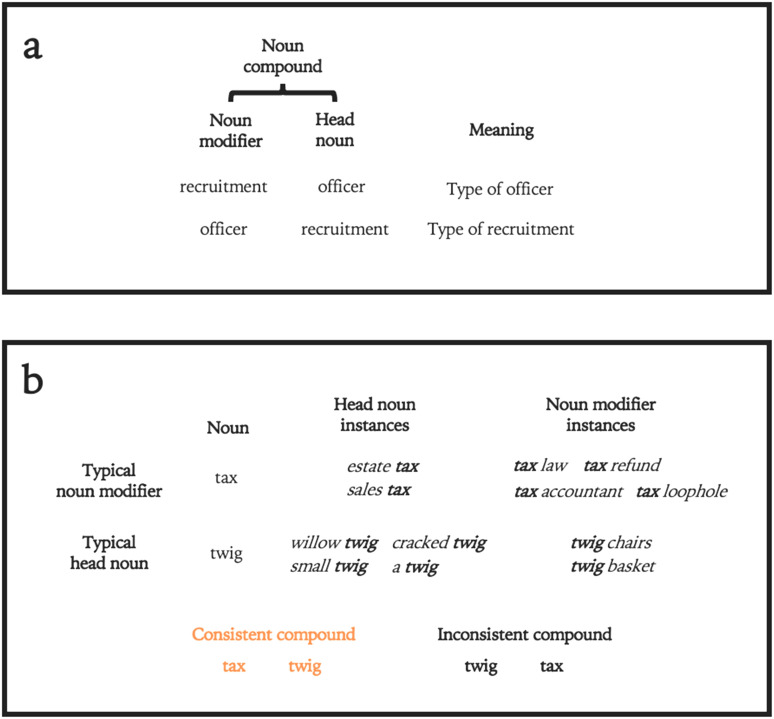
**(A) Anatomy of a noun compound.** In English, noun compounds are composed of a noun modifier followed by a head noun. In natural language, nouns may occur as both noun modifiers and head nouns, and order affects meaning, as illustrated with *recruitment* and *officer*. **(B) Creating novel noun compounds.** Some nouns (e.g., *tax*) are more commonly noun modifiers than others (*twig*). In these experiments, consistent compounds contained a typical noun modifier followed by a typical head noun; inconsistent compounds reversed this order.

As shown in Figure 1, we grouped nouns into pairs of novel noun compounds, in which a noun modifier preceded a head noun. Any noun can be a head noun or noun modifier (Figure 1A), but there are strong statistical patterns of noun modifier usage in English, which affect comprehension (MacDonald, 1993). To investigate whether these statistics affect VWM performance, we presented participants with two noun orders (Figure 1B). Consistent pairs followed the statistics of English; a typical noun modifier preceded a typical head noun. Inconsistent pairs reversed these words, so that the two conditions were identical except for the order of the critical words. If Consistent novel noun pairs yield superior performance than Inconsistent ones, this result would favor emergent accounts of VWM driven by language experience.

Experiments 1–2 used these materials in immediate serial recall, and Experiment 3 employed a recognition task. Experiments 2–3 included a language experience measure (Acheson et al., 2008); emergent VWM accounts predict that language experience enriches linguistic LTM, with benefits for VWM performance.

## EXPERIMENTS 1 AND 2

### Methods

#### Participants.

Given the novelty of our manipulation, in which the Consistent and Inconsistent conditions are identical save for the order of two words, it was impossible to estimate effect sizes or power *a priori*. We chose a target sample size of 100 for Experiment 1 and 150 for Experiment 2, which contained an individual differences component. Analysis of individual differences increases model complexity in language (e.g., Farmer et al., 2017), so that additional participants were warranted.

A total of 108 UW-Madison undergraduates took part in Experiment 1 and 159 participated in Experiment 2. A few extra participants were tested in each experiment in case some needed to be removed because of equipment failure or other reasons. None were removed from Experiment 1. Five participants were excluded from Experiment 2: three for computer errors, one for the production of inaudible responses, and one for reporting difficulty attending and responding during the task, leaving 154 participants in final analyses (*M*_age_ = 18.60; 93 female). Due to a collection error, age and gender of participants in Experiment 1 are not available. No analyses, including descriptive statistics, were conducted until sampling was finished.

All participants were native speakers of English, participated for course credit, and provided informed consent according to the guidelines established by the University of Wisconsin-Madison IRB.

#### Materials.

To develop critical word pairs for the memory lists, usage patterns of typical noun modifiers and typical head nouns were identified in the Corpus of Contemporary American English (COCA; Davies, 2008). Selection methods and criteria for selecting list words are described in the supplemental materials. By virtue of the different ways that they are used in language, common noun modifiers and head nouns necessarily have different patterns of usage in natural language. According to a *t* test of the log probability of the stimuli in the SUBTLEXus corpus (Brysbaert & New, 2009), head nouns employed in Experiment 1 were more frequent than noun modifiers employed in Experiment 1, *t*(1, 158) = 2.15, *p* < .05. In Experiment 2, which used completely different critical pairs, head nouns and noun modifiers did not differ in frequency, *t*(1, 158) = 1.60, *p* > .05. Distributions of the frequencies of the nouns can be viewed in Figure S.1.1.

Following selection of typical noun modifiers and typical head nouns in each experiment, these words were paired together, forming sets of critical pairs. Critical pairs were generated such that the two nouns never co-occurred in COCA within a window of 4 words. Pairings were randomly sampled in each study until a total of 80 critical pairs were generated. Two different sets of nouns were selected for Experiment 1 and Experiment 2, to provide a replication and extend the generalizability of the results across items.

Critical pairs and fillers were presented to participants in a recall task using 6-word lists. In each experiment, words in positions 3 and 4 were drawn from the list of critical pairs for that study. Trials took one of two forms. In the Consistent condition (i.e., consistent with the word usage patterns of English), word 3 was a typical noun modifier and word 4 was a typical head noun. In the inconsistent condition, these words were reversed, with the typical head noun appearing in position 3 and the typical noun modifier appearing in position 4. Condition (consistent, inconsistent) was randomized for each trial, with participants seeing an equal number of consistent and inconsistent conditions.

**Figure 2.**
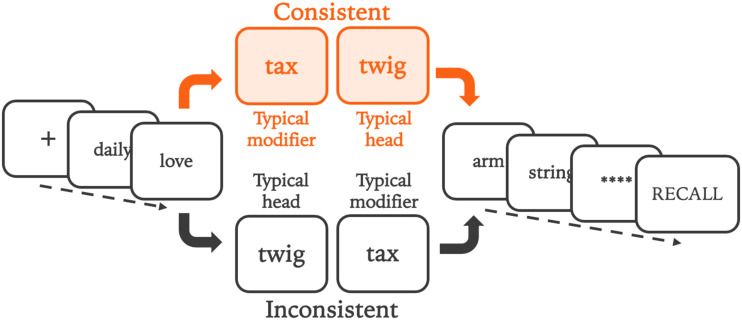
**Format of the serial recall lists in Experiments 1–2.** Consistent or inconsistent critical pair in word positions 3 and 4. This figure uses color, but participants saw all materials in black font on a white background. Filler words shown in this figure are a random selection, as in Experiment 1. See method for the more constrained selection of fillers in word positions 2 and 5 in Experiment 2.

Words in positions surrounding the critical pair (positions 1, 2, 5, 6) were chosen randomly from the set of filler words in Experiment 1. The random assignment of filler words for each trial and each participant would be expected to control for any relationships between the words in the critical pair and the adjacent words. In Experiment 2 we added extra controls to the fillers to further reduce any chances that critical pair words might be integrated with fillers. In Experiment 2, a randomly selected plural noun always appeared in position 2, just before the critical pair, because a plural noun is unlikely to be a modifier of an upcoming noun phrase. This choice reduced the likelihood of any relationships between the filler word in position 2 and the first element of the critical pair in position 3. Similarly, a randomly selected adjective always appeared in position 5, after the critical pair; an adjective is not likely to integrate with a prior noun. Words in position 1 and 6 were randomly sampled fillers, as in Experiment 1. A summary of the list structure for both experiments is presented in Figure 2.

In both studies, the order of critical pairs across trials was randomized for each participant. Each filler word and critical pair was presented only once to each participant across an experiment.

#### Procedure.

Participants were instructed that they would participate in a memory experiment in which they were to recall words in the same order that they were presented. Participants first completed five practice trials after which they were provided the opportunity to ask a research assistant for clarification about the task. Following completion of the practice, the door to the participant’s sound-proof, individual testing room was closed, and participants progressed through the task at their own pace.

The timing of stimulus presentation varied slightly in Experiment 1 and 2. We made the list presentation quite rapid in Experiment 1 to discourage any deliberate integration of words in the list. As post-experiment reports suggested that this was not a concern in Experiment 1, presentations were lengthened in Experiment 2 to be more similar to durations in other immediate serial recall studies.

At the beginning of each trial, participants saw a fixation cross for 250 ms (Experiment 1) or 350 ms (Experiment 2). Each word was presented for 600 ms (Experiment 1) or 800 ms (Experiment 2) with a 50 ms interstimulus interval in both studies. Following the presentation of all list words, a visual buffer (‘****’) was displayed for 300 ms, and the word ‘RECALL’ was displayed on screen in both studies. Participants spoke aloud when recalling words. The RECALL prompt remained on the screen, and participant responses were recorded until participants pressed a key to end the current trial and move to the next.

The only other difference between Experiments 1–2 was that the Author Recognition Test (ART; Acheson et al., 2008) was administered to participants at the beginning of the Experiment 2, before the recall task. This measure of reading experience was chosen as an assessment of language experience because reading rates are more likely than spoken language usage to vary in the college student population, and because the ART has been shown to predict patterns of both language comprehension (Acheson et al., 2008) and language production (Montag & MacDonald, 2015). In the ART, participants were presented with several grids of author and foil names. Participants were tasked with clicking on names that they were sure were real authors and ignoring non-author names. Scoring gave credit for real authors identified minus foil authors that were selected.

Following completion of the recall task, participants were asked debriefing questions probing their strategies, any intuitions about patterns in the stimuli, and hypotheses about the purpose of the experiment. No participant in either study reported recognizing real patterns in the lists, nor did any participant report using strategies grouping words based on their grammatical relations.

### Results

**Figure 3.**
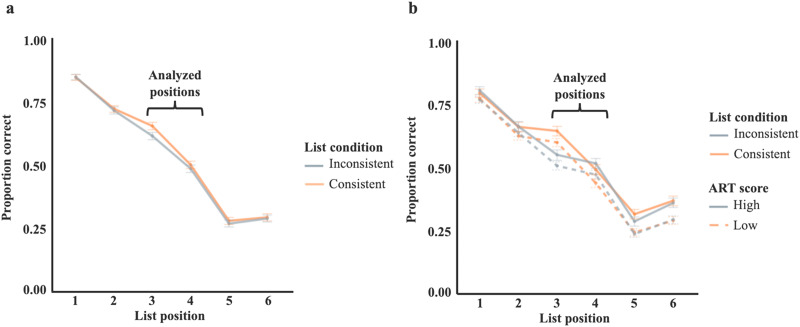
**Performance in Experiment 1 and Experiment 2.** **(A)** Proportion correct in position across list position for Experiment 1. Bars represent standard error of the mean bootstrapped from raw data. Analyzed positions correspond to positions 3 and 4; other list positions are provided for context. **(B)** In Experiment 2, the high vs low ART is dichotomized for the purpose of visualization. In analyses, ART was analyzed as a continuous variable.

Accuracy in recalling words in the critical pair in position were fit using a binomial mixed effects logistic regression, described in detail in the supplemental materials. Fixed effects included consistency of the compound, list position, and their interaction. Recall was marked as correct if the word was recalled in the position in which it was presented. Recalling a word in any other position or omitting a word was marked as incorrect. Performance in both experiments is summarized in Figure 3.

Despite the use of different list materials in the two experiments, the results were similar. In Experiment 1, participants had significantly higher recall accuracy for the Consistent compared to the Inconsistent pairs, as indicated by a main effect of list condition, *b* = 0.13, *X*^2^(1) = 8.10, *p* = .004. Participants were also significantly better at recalling words in position 3 than position 4, *b* = −0.68, *X*^2^(1) = 197.46, *p* < .001. There was no interaction between condition and position, *X*^2^(1) = 1.03, *p* = .31, resulting in higher recall for consistent over inconsistent words in position 3 (*m*_con_ = .65, *m*_incon_ = .61) as well as position 4 (*m*_con_ = .50, *m*_incon_ = .48).

In Experiment 2, participants again were significantly better at recalling words presented in the Consistent than the Inconsistent word order, *b* = 0.15, *X*^2^(1) = 21.37, *p* < .001. Again, there was an effect of position, such that words in position 3 were recalled better than words in position 4, *b* = −0.45, *X*^2^(1) = 93.70, *p* < .001. Unlike in Experiment 1, the effect of position interacted with list condition, such that the difference between Consistent and Inconsistent orders was greater in position 3 than in position 4, *b* = −0.58, *X*^2^(1) = 18.58, *p* < .001. The effect of condition was numerically reversed in position 4 (*m*_con_ = .45, *m*_incon_ = .48) compared to position 3 (*m*_con_ = .60, *m*_incon_ = .51), resulting in higher recall in the inconsistent condition compared to the consistent condition in position 4.

Also in Experiment 2, there was a main effect of participants’ ART score on recall; participants with higher ART scores were better at recalling the critical pair words, *b* = 0.03, *X*^2^(1) = 5.65, *p* = .017. There was no interaction between list condition and ART score, *X*^2^(1) = 3.60, *p* = .058.

### Discussion

In both studies, subtle language statistics affected recall in a VWM task in which conditions differed only by the order of two nouns. These results show that linguistic LTM affects VWM, even for novel noun sequences, consistent with emergent theories that do not posit a separate temporary store (Schwering & MacDonald, 2020).

The interaction between consistency and position, reliable in Experiment 2 only, merits future research. We offer one post-hoc explanation: consistent contexts may have a larger effect on noun modifiers in position 3 compared to head nouns, because noun modifiers are present only in noun compounds, while head nouns occur in a variety of contexts. These sorts of interactions between words and context guide comprehension (MacDonald et al., 1994), and this result may suggest these interactions also affect VWM. Nevertheless, the fact that this interaction was not present in Experiment 1 suggests either differences in the specific items used in Experiments 1 and 2, differences between participants in the samples, the subtle differences in experimental designs, or random chance may have moderated the interaction among the compound context and the noun modifier and head noun. Future research could further examine the qualities of the modifiers and heads that influence the goodness of the noun compounds.

Future research could also consider the extent to which the influence of noun compound context on VWM depends on recall of constituents of the noun compound. Prior research suggests that participants may access abstract syntactic structures without accessing particular words during sentence reconstruction tasks (Lombardi & Potter, 1992; Potter & Lombardi, 1990). If similar mechanism(s) are at play in the recall of noun compounds, explicit recall of one constituent of the compound may not depend on recall of the other, so long as the higher order structure of the noun compound was encoded during study. In this way, language comprehension processes may play an important role in encoding linguistic memoranda.

Language experience, measured via ART scores, also predicted recall in Experiment 2, again suggesting that language experience supports performance in VWM tasks. ART scores did not interact with the consistency manipulation. ART performance does predict performance on complex syntactic structures in comprehension (Acheson et al., 2008) and production tasks (Montag & MacDonald, 2015), but noun compounds are extremely frequent in English, and sensitivity to them is not likely to vary within our college student population.

In Experiment 3, we extended Experiments 1 and 2 by examining the ways in which noun compound consistency affects order recognition (preregistration analysis: https://osf.io/4m6vz/?view_only=63abdf2a5f454942a5bb1f48d29fb2cb; Foster & Deardorff, 2017). In this task, we tested memory over a longer time scale compared to the serial recall tasks employed in Experiments 1 and 2. Our order recognition task is presented several minutes after initial presentation of the noun compounds, and the order recognition task can therefore be considered an episodic memory task rather than strictly a VWM task. We nonetheless believe this longer time scale allows us to test the generalization of the effects of noun compound consistency on memory at longer delays and into the domain of episodic memory (Baddeley, 2000; Ranganath, 2010; Yonelinas, 2001).

The order recognition task also has several theoretical benefits regarding the integration of word and word order representations. Serial recall and strict serial scoring measure a composite of memory items and their order (Saint-Aubin & Poirier, 1999), and researchers use order recognition and reconstruction tasks to distinguish order and item memory. Given the essential integration between words and orders in language processing, rich emergent approaches have argued that linguistic LTM should also support VWM for both words and orders (Schwering & MacDonald, 2020). Experiment 3 provides a test of this claim: if LTM of noun compound statistics affects memory for the order of words, then Consistent pairs should be rated as old more than Inconsistent pairs.

This task has three additional benefits. First, the recognition task assesses memory of the critical pair directly, obviating the possibility that better recall of one constituent drives the observed effect of consistency. Second, recognition requires no overt articulation, removing overt production as an explanation of effects. Third, we measured meaningfulness of the critical pairs in the study phase, allowing us to disentangle pair meaningfulness and our noun statistics manipulation.

## EXPERIMENT 3

### Methods

#### Participants.

A total of 140 UW-undergraduates participated. Sample size followed preregistration of analysis guidelines (https://osf.io/4m6vz/?view_only=63abdf2a5f454942a5bb1f48d29fb2cb), with sample collection finishing as preregistration of analysis was submitted. Following application of pre-registered exclusion criteria, 129 participants remained in analyses (*M*_age_ = 18.4; 61 female). All remaining participants were native speakers of English.

#### Materials.

All noun compounds from Experiments 1 and 2 were used in this experiment. The ART materials (Acheson et al., 2008) were also used.

#### Procedure.

**Figure 4.**
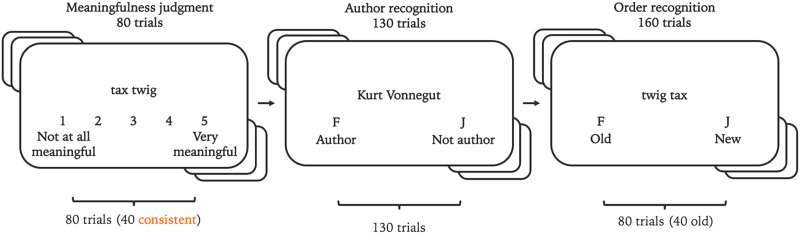
**Sequence of tasks in Experiment 3.** Participants used the numerals 1–5 on a keyboard to make meaningfulness judgments in the first task, and they used the F key and the J key to make judgments in the Author Recognition Task (ART) and the order recognition task.

Following completion of informed consent and a demographics survey, participants completed three tasks. A summary of the procedure is shown in Figure 4.

##### *Compound Judgment Task*.

In the first task, participants were asked to rate noun compounds for meaningfulness using a 1–5 scale. Because presenting the full set of 160 word pairs from Experiments 1–2 would have resulted in an overly-long experiment, each participant was presented a random subset of 80 noun compounds from the pool of 160 developed for Experiment 1 and Experiment 2. Word pairs were presented one at a time on a computer screen, either in the order consistent with prior grammatical experience (i.e., typical noun modifier followed by typical head noun) or in an inconsistent order (i.e., typical head noun followed by typical noun modifier). Each participant saw 40 pairs in the consistent order and 40 pairs in the inconsistent order. Order for each pair was randomized.

##### *Author Recognition Task*.

In the second task, participants completed the Author Recognition Test (Acheson et al., 2008). Participants saw one name at a time and pressed a key to indicate whether the name was an author or not.

##### *Compound Recognition*.

In the third task, participants were presented with the same noun compounds presented in the compound judgment. Noun compounds were presented either in the same order as was shown in the meaningfulness judgment task or in the opposite order. Participants were informed that all words in the pairs had been presented previously, and they were instructed to judge specifically whether the word order of the compound was old (presented previously) or new.

### Results

In the first model, participants’ old responses were predicted from oldness of the word order, consistency of the pair with language statistics, ART score, and the interaction between ART score and the two other factors. Participants were more likely to indicate a pair order was old if the pair order was actually old, as indicated by a significant effect of oldness of the pair order, *b* = 1.39, *X*^2^(1) = 239.42, *p* < .001. Consistent with the preregistered predictions of our language emergent account, participants were more likely to indicate a pair was old if the noun order was Consistent compared to Inconsistent, *b* = 0.47, *X*^2^(1) = 46.03, *p* < .001. This result shows sensitivity to statistical patterns in VWM tasks even without overt language production.

**Figure 5.**
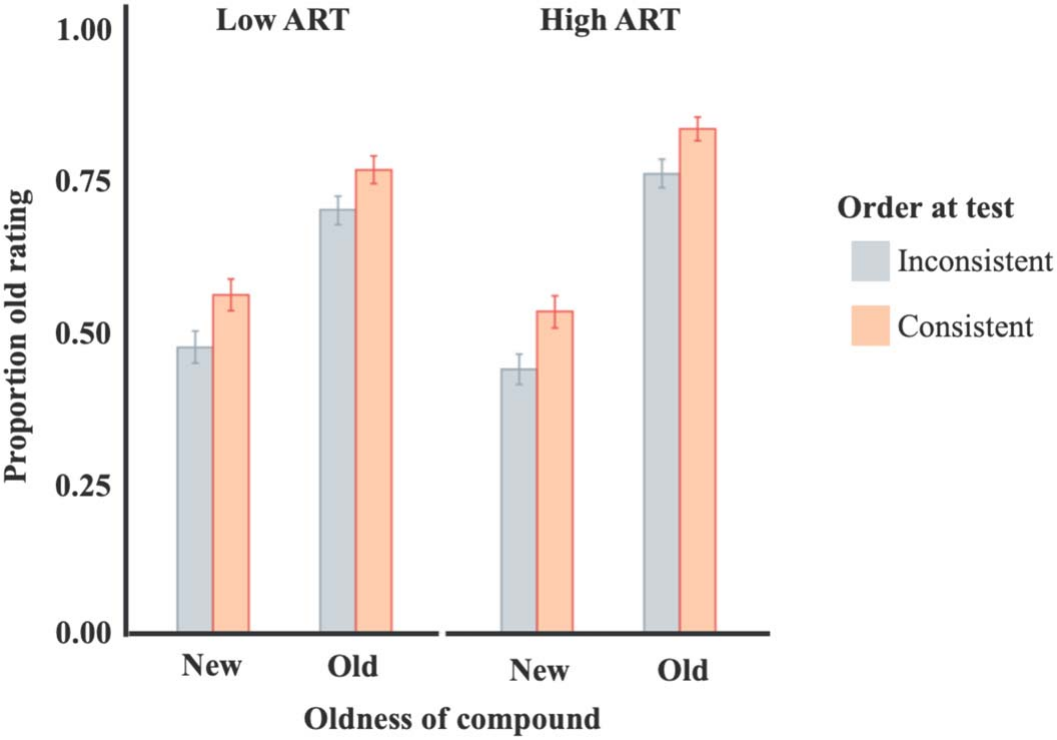
**Performance in Experiment 3.** Proportion old responses in order recognition task for Experiment 3. Error bars represent standard error bootstrapped from raw data. ART scores were dichotomized for display in this figure.

Participant ART score was not a significant predictor of overall rates of old ratings, *X*^2^(1) = 1.13, *p* = .29. However, ART score did interact with age of the pair order such that old pairs were more likely to be correctly judged as old by participants with higher ART scores, *b* = 0.04, *X*^2^(1) = 7.14, *p* < .01. Similar to the results of Experiment 2, there was no interaction between participant ART score and consistency of the pair constituents with their typical grammatical roles, *X*^2^(1) = 0.01, *p* = .93. Performance is summarized in Figure 5.

An additional model was fit including each participant’s meaningfulness rating as a covariate. The judgment of meaningfulness predicted old rating, such that higher ratings of meaningfulness corresponded to more old ratings, *b* = 0.07, *X*^2^(1) = 11.23, *p* < .001. A key question is therefore whether our Consistent/Inconsistent word order manipulation is simply a proxy for meaningfulness of the pairs, or whether both meaningfulness and word order statistics both predict order recognition behavior. If the effects are driven entirely by meaningfulness, then there should be no effect of our statistics-based consistency manipulation once meaningfulness is in the model, and performance on our items could simply be ascribed to chunking the pairs that were more meaningful. Analyses did not support this meaningfulness-based reinterpretation of our results. Participants were still more likely to indicate that a pair order was old if the pair order conformed to long-term statistical patterns dictated by the constituents, *b* = 0.47, *X*^2^(1) = 46.35, *p* < .001, even when controlling for the meaningfulness rating of the original compound.

## GENERAL DISCUSSION

In two experiments, we investigated whether subtle noun statistics that affect language comprehension also shape VWM performance, as predicted by theories of emergent VWM (Schwering & MacDonald, 2020). Additionally, in a third experiment, we explored the ways in which language processes can shape order recognition memory at longer timescales. In Experiments 1–2, order of nouns in novel pairs reliably affected recall in otherwise identical lists, showing the importance of language statistics in VWM. Experiment 3 demonstrated a similar pattern of results, in which memory consistent compounds were more likely to be rated as old than inconsistent noun compounds (Schwering & MacDonald, 2020). Our order manipulation affected order memory even after considering meaningfulness of the novel word pairs, showing for the first time that subtle noun order statistics driving everyday language use also support order recognition memory over a long delay. Experiments 2–3 also showed that a measure of language experience predicted VWM performance, further supporting a role for linguistic LTM on memory tasks.

This work informs theories of VWM in several ways. First, these data invite reconsideration of sentence superiority effects. Some accounts of the effect have pointed to the meaningfulness of sentence-like memory lists, suggesting that participants are grouping list items into meaningful chunks, based on their prior occurrence and/or association (Jones & Farrell, 2018). Another interpretation holds that participants chunk list items from part-of-speech statistics (Jones & Farrell, 2018; Perham et al., 2009). Neither explanation holds for our findings. Our noun pairs had no part-of-speech variation, and we showed that pair meaningfulness could not account for our noun order effects. If a chunking explanation were offered for our results, it would effectively be driven by the linguistic LTM of subtle noun statistics beyond part-of-speech and the functional relationship of the head and modifier of the compound. That is the essence of the rich emergent approach to VWM; “chunking” is LTM-guided language processing.

Second, these results invite further consideration of novelty in theorizing in VWM, because they suggest that language processes can contribute significantly to encoding and maintenance of novel noun sequences. Our research argues against claims that sequences in memory lists are so novel that LTM cannot support VWM performance (Norris, 2017). Future research could employ continuous measures of sentence-likeness, such as acceptability ratings (Sprouse et al., 2018), to measure whether linguistic LTM continuously bears on memory for typical lists. Findings of VWM capacity continuously scaling with sentence-likeness would suggest language comprehension and production mechanisms are intimately engaged with VWM. This approach would address limitations in the current research, expanding beyond noun pairs and the necessarily subtle effects of our highly controlled manipulation.

These results suggest that word representations and word order interact to inform VWM. We have previously discussed the importance of this interaction in the rich emergent model of VWM (Schwering & MacDonald, 2020). While we believe these interacting representations are important for language use and VWM, our current results cannot determine whether the language system affects both item and order memory. Previous research has suggested that language comprehension and production mechanisms may support item memory—memory for previously encountered words—but not order memory—memory for the order in which those words were encountered. Experiment 3 provides tentative evidence that the order of noun compounds affects order memory, though the design of this experiment differs in several ways from previous tests of order memory (e.g., Majerus et al., 2015; Nairne & Kelley, 2004). The delay between study and test in Experiment 3 may reflect some contribution of episodic memory to task performance. However, the effects of linguistic LTM in Experiment 3, that consistent pairs were rated “old” more than inconsistent pairs, shows that for these verbal materials, linguistic LTM is influencing performance in this recognition task, consistent with previous studies (Jacobs et al., 2016) measuring the impact of linguistic representations on recognition.

Additionally, this work offers a new methodological approach compared to prior methods investigating the interaction between language processes and VWM. Our corpus analyses provide a precise way to measure linguistic LTM while controlling the co-occurrence associations among words. Further, the specific manipulation, in which the same words are employed between conditions but in a different order, allow us to examine how the interaction among lexical and syntactic representations influence VWM. Previous studies of the sentence superiority effect have used scrambled lists (e.g., Allen et al., 2018), but in contrast to prior work, all conditions of our experiments form valid noun compounds. Because all materials are both grammatical and are novel sequences, this design allows us to examine how the degree of similarity between our stimuli and natural language influence VWM in a graded way.

Finally, this work informs how VWM tasks should be interpreted in psycholinguistics, child development, and aging and impairment. Findings that VWM tasks are better characterized as assessments of language knowledge or skill (e.g., Klem et al., 2015) gain credence with our demonstrations that language-based manipulations affect VWM. This work also supports skepticism of the benefits of working memory training (Melby-Lervåg & Hulme, 2013), because the VWM is not a language-independent system that can be trained from repeated VWM tasks. Instead, improvement would likely come from additional language experience, strengthening linguistic LTM and language processes that support VWM.

## AUTHOR CONTRIBUTIONS

Authors SS and MM contributed to the design of all 3 experiments and preparation of the manuscript. Author SS conducted analyses.

## FUNDING INFORMATION

This research was funded by NSF grant #1849236, the NSF funded UW-Madison Psychology PREP program, Wisconsin Alumni Research Foundation (WARF) awards, and the Menzies and Royalty Research Award at the University of Wisconsin-Madison.

## DATA AVAILABILITY STATEMENT

All data and analyses are available on OSF (https://osf.io/yqesf/).
