# Supplementary material for: Noun Sequence Statistics Affect Serial Recall and Order Recognition Memory
Source: Open Mind (Camb). 2023 Aug 11;7:550–63. doi: 10.1162/opmi_a_00092 (PMC10449402; doi:10.1162/opmi_a_00092)
Supplement: Supplementary file 1 [file opmi-07-550-s001.docx]

**Supplementary Materials**

All analysis can be found in our publicly available datasets and analysis scripts (https://osf.io/yqesf/?view_only=f0d2f0ee65e6451baeb0ff01aa71b7b1). Information regarding the preregistration of Experiment 3 can also be found on OSF (<https://osf.io/4m6vz/?view_only=63abdf2a5f454942a5bb1f48d29fb2cb>).

**S1. Stimulus selection**

**S1.1. Stimulus selection of Experiment 1.**

*Typical noun modifier*. Total frequency of a typical noun modifier was determined by searching the COCA web interface for the word string and recording the total number of occurrences (search: “<WORD>”). The relative probability of that word occurring as a noun modifier was calculated by searching for the frequency with which that word occurred immediately before any noun (search: “<WORD> NOUN”). This frequency was divided by the total frequency of the word, giving an estimate of the probability of the word occurring as a noun modifier across all contexts. Frequency and probability of noun modifiers employed in Experiment 1 can be found in *Table S1.1.*

*Typical head nouns*. Total frequency was again calculated by searching for a word string and recording the total number of occurrences (search: “<WORD>”). The frequency with which that word occurred as a head noun was then calculated by subtracting the frequency with which that word occurred as a verb (search: “<WORD>_v*”) and the frequency with which the word occurred before another noun (search: “<WORD> NOUN”) from the total frequency of the word. This frequency as a head noun was then divided by the total frequency of the word to provide an estimate of the probability of the word occurring as a head noun across all contexts. Frequency and probability of noun modifiers employed in Experiment 1 can be found in *Table S1.2.*

*Filler words*. Filler words were selected from a variety of grammatical roles to have comparable length and frequency to the critical nouns.

**Table S1.1. Noun compounds in Experiment 1**.

| Noun modifier | Head noun | Modifier freq. as modifier | Head freq. as head | Modifier prob. as modifier | Head prob. as head |
| --- | --- | --- | --- | --- | --- |
| human | ducks | 140035 | 4654 | .864 | .983 |
| peanut | writer | 4690 | 32151 | .857 | .984 |
| auto | flag | 11785 | 12853 | .838 | .863 |
| civilian | suggestion | 13837 | 8017 | .810 | .990 |
| criminal | grandmother | 28609 | 16763 | .794 | .984 |
| bowel | pillow | 1329 | 5963 | .770 | .899 |
| chemical | couch | 21125 | 11576 | .742 | .938 |
| plastic | athlete | 30867 | 8957 | .738 | .908 |
| carbon | soldier | 15600 | 14835 | .730 | .924 |
| silicon | farmer | 5648 | 9143 | .725 | .955 |
| drug | sailor | 64518 | 3131 | .716 | .910 |
| rubber | flavor | 9073 | 11454 | .701 | .850 |
| tourist | spade | 7414 | 1130 | .698 | .897 |
| lung | actor | 5991 | 20053 | .690 | .961 |
| emergency | tray | 26216 | 5950 | .689 | .950 |
| military | jar | 119705 | 5597 | .680 | .921 |
| web | prince | 43115 | 17954 | .670 | .981 |
| leather | argument | 14715 | 29284 | .664 | .990 |
| tax | twig | 76422 | 903 | .658 | .845 |
| immigrant | creator | 9902 | 4858 | .652 | .974 |
| kidney | crowd | 4581 | 34320 | .647 | .913 |
| health | hat | 183880 | 19759 | .643 | .954 |
| passenger | character | 9878 | 42947 | .636 | .907 |
| aluminum | finger | 8040 | 21131 | .632 | .909 |
| nylon | ear | 2470 | 20464 | .631 | .851 |
| railway | skirt | 2412 | 8420 | .625 | .898 |
| potato | hunter | 8240 | 12360 | .621 | .919 |
| cardboard | logic | 4666 | 10631 | .609 | .931 |
| metal | journalist | 26984 | 10053 | .605 | .978 |
| motor | plumber | 14826 | 1006 | .604 | .976 |
| tomato | blade | 8530 | 9229 | .592 | .902 |
| golf | clerk | 26954 | 7081 | .591 | .980 |
| diesel | advisor | 3994 | 3003 | .587 | .965 |
| cable | poem | 19915 | 10594 | .580 | .983 |
| banana | secretary | 4462 | 47277 | .572 | .981 |
| silk | exam | 9183 | 5236 | .571 | .849 |
| bronze | uncle | 6216 | 22424 | .567 | .985 |
| brick | wrist | 8977 | 7814 | .565 | .865 |
| brass | maid | 6543 | 3867 | .564 | .908 |
| soccer | hole | 12485 | 27687 | .557 | .958 |
| insect | girl | 4179 | 64071 | .550 | .959 |
| pet | texture | 9459 | 6302 | .544 | .965 |
| cotton | society | 13979 | 89991 | .543 | .955 |
| football | badge | 35977 | 2928 | .542 | .922 |
| computer | vest | 63135 | 3197 | .537 | .942 |
| associate | acoustics | 14925 | 606 | .536 | .932 |
| video | fork | 57308 | 8203 | .533 | .923 |
| rib | lamp | 2405 | 6031 | .526 | .907 |
| slave | level | 8098 | 113008 | .524 | .934 |
| iron | lawyer | 19339 | 30674 | .523 | .969 |
| basketball | plate | 26040 | 22306 | .523 | .926 |
| clam | manager | 1070 | 41003 | .521 | .978 |
| stem | beer | 12640 | 23348 | .508 | .859 |
| linen | plane | 3604 | 31411 | .506 | .897 |
| insurance | yard | 44919 | 19395 | .503 | .849 |
| wool | giants | 5714 | 11534 | .502 | .904 |
| ice | wallet | 43299 | 5042 | .498 | .972 |
| backup | bed | 5797 | 68999 | .485 | .958 |
| picnic | position | 4770 | 78103 | .484 | .945 |
| chocolate | letter | 16052 | 45245 | .481 | .936 |
| jazz | desk | 13640 | 28545 | .466 | .915 |
| gold | thunder | 42161 | 4598 | .466 | .852 |
| flower | motion | 10639 | 20696 | .464 | .843 |
| coal | device | 10721 | 18372 | .462 | .942 |
| net | theory | 20726 | 46726 | .459 | .975 |
| mountain | stranger | 41090 | 10248 | .448 | .940 |
| square | shrine | 37556 | 3268 | .445 | .923 |
| crystal | agency | 9674 | 47138 | .436 | .903 |
| college | mist | 122156 | 4638 | .436 | .906 |
| appliance | addition | 1353 | 56958 | .431 | .997 |
| apple | explosion | 19467 | 10215 | .425 | .981 |
| pancake | seat | 1418 | 40675 | .417 | .893 |
| ant | afternoon | 2181 | 39907 | .415 | .878 |
| chicken | boat | 29668 | 30543 | .413 | .847 |
| cooking | driver | 23484 | 29471 | .408 | .951 |
| pickle | gate | 1036 | 15991 | .402 | .842 |
| math | pocket | 16307 | 21795 | .395 | .860 |
| safety | pen | 46775 | 9231 | .391 | .867 |
| desert | corporation | 20543 | 11691 | .385 | .950 |
| garbage | equation | 8661 | 7535 | .382 | .943 |

**Table S1.2. Noun modifier and head noun SUBTLEXus frequencies in Experiment 1.**

| Noun modifier | Modifier SUBTLEXus log frequency | Head noun | Head SUBTLEXus log frequency |
| --- | --- | --- | --- |
| human | 3.80 | ducks | 2.64 |
| peanut | 2.80 | writer | 3.08 |
| auto | 2.59 | flag | 2.95 |
| civilian | 2.68 | suggestion | 2.79 |
| criminal | 3.25 | grandmother | 3.15 |
| bowel | 2.26 | pillow | 2.76 |
| chemical | 2.76 | couch | 3.08 |
| plastic | 2.98 | athlete | 2.37 |
| carbon | 2.43 | soldier | 3.30 |
| silicon | 1.94 | farmer | 2.78 |
| drug | 3.36 | sailor | 2.80 |
| rubber | 2.85 | flavor | 2.41 |
| tourist | 2.38 | spade | 2.08 |
| lung | 2.62 | actor | 3.13 |
| emergency | 3.44 | tray | 2.61 |
| military | 3.32 | jar | 2.63 |
| web | 2.67 | prince | 3.36 |
| leather | 2.84 | argument | 2.94 |
| tax | 2.87 | twig | 1.85 |
| immigrant | 1.90 | creator | 2.16 |
| kidney | 2.69 | crowd | 3.28 |
| health | 3.31 | hat | 3.52 |
| passenger | 2.74 | character | 3.29 |
| aluminum | 2.06 | finger | 3.27 |
| nylon | 1.85 | ear | 3.21 |
| railway | 1.91 | skirt | 2.71 |
| potato | 2.76 | hunter | 2.97 |
| cardboard | 2.14 | logic | 2.54 |
| metal | 3.00 | journalist | 2.49 |
| motor | 2.83 | plumber | 2.36 |
| tomato | 2.48 | blade | 2.82 |
| golf | 3.11 | clerk | 2.82 |
| diesel | 2.13 | advisor | 2.35 |
| cable | 3.04 | poem | 2.84 |
| banana | 2.74 | secretary | 3.23 |
| silk | 2.70 | exam | 2.84 |
| bronze | 2.16 | uncle | 3.80 |
| brick | 2.72 | wrist | 2.72 |
| brass | 2.79 | maid | 3.07 |
| soccer | 2.81 | hole | 3.47 |
| insect | 2.21 | girl | 4.45 |
| pet | 3.01 | texture | 1.93 |
| cotton | 2.86 | society | 3.23 |
| football | 3.28 | badge | 2.89 |
| computer | 3.48 | vest | 2.45 |
| associate | 2.66 | acoustics | 1.36 |
| video | 3.32 | fork | 2.65 |
| rib | 2.48 | lamp | 2.82 |
| slave | 2.97 | level | 3.42 |
| iron | 2.96 | lawyer | 3.61 |
| basketball | 3.04 | plate | 3.12 |
| clam | 2.30 | manager | 3.31 |
| stem | 2.06 | beer | 3.59 |
| insurance | 3.29 | yard | 3.11 |
| linen | 2.18 | plane | 3.69 |
| wool | 2.21 | giants | 2.35 |
| ice | 3.61 | wallet | 3.07 |
| backup | 2.94 | bed | 3.98 |
| picnic | 2.78 | position | 3.57 |
| chocolate | 3.18 | letter | 3.62 |
| jazz | 2.78 | desk | 3.35 |
| gold | 3.61 | thunder | 2.83 |
| flower | 3.07 | motion | 2.99 |
| coal | 2.53 | device | 2.97 |
| net | 2.9 | theory | 3.16 |
| mountain | 3.26 | stranger | 3.14 |
| square | 3.21 | shrine | 2.18 |
| crystal | 2.92 | agency | 3.00 |
| college | 3.64 | mist | 2.26 |
| appliance | 1.62 | addition | 2.60 |
| apple | 3.08 | explosion | 2.93 |
| pancake | 2.31 | seat | 3.60 |
| ant | 2.44 | afternoon | 3.67 |
| chicken | 3.50 | boat | 3.69 |
| cooking | 3.12 | driver | 3.38 |
| pickle | 2.37 | gate | 3.21 |
| math | 2.92 | pocket | 3.26 |
| safety | 3.22 | pen | 3.10 |
| desert | 3.15 | corporation | 2.60 |
| garbage | 3.12 | equation | 2.25 |

***Figure S1.1.* Frequencies of noun modifiers and head nouns in Experiment 1 and 2.**

**
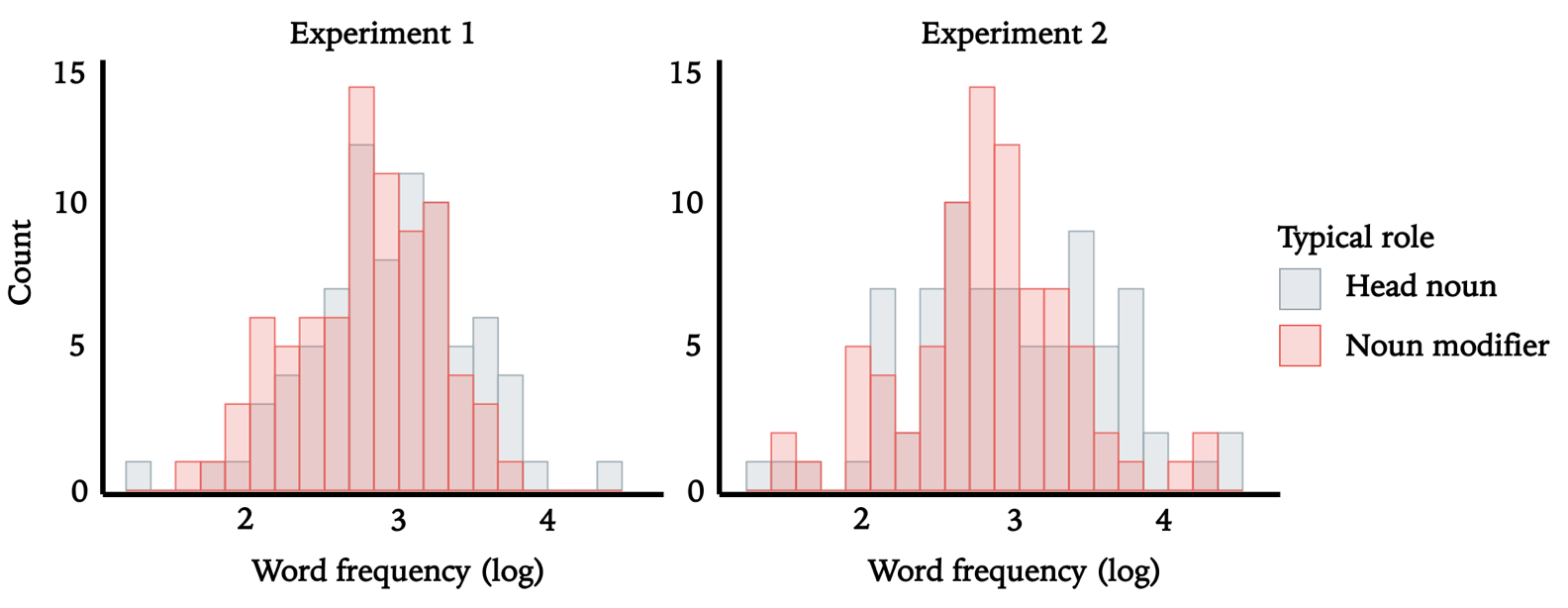
**

**S1.2. Stimulus selection of Experiment 2.**

Noun modifiers and head nouns were identified and paired by calculating the probabilities in grammatical roles over all nouns in the part of speech tagged COCA corpus. This was performed by a series of Python scripts written by the first author. All nouns and their immediate contexts were extracted from the corpus and analyzed to select new pairs. Final lists of six words were generated randomly such that critical pairs always appeared in positions 3 and 4.

*Noun modifiers*. An occurrence of a noun was identified as a noun modifier if it had a part-of-speech tag as a noun and occurred immediately before another noun. The total frequency in the noun modifier role was then divided by the total frequency of a word to provide the probability of that word occurring as a noun modifier. Frequency and probability of noun modifiers employed in Experiment 2 can be found in *Table S1.3.*

*Head nouns*. An occurrence of a noun was identified as a head noun if it had a part-of-speech tag as a noun and did not occur immediately before another noun. The total frequency of a word occurring in the role of a head noun was then divided by the total frequency of that word to provide the probability of that word occurring as a head noun. Frequency and probability of head nouns employed in Experiment 2 can be found in *Table S1.3.*

*Critical pairs*. Critical pairs were generated such that noun modifiers and head nouns did not co-occur within a window of 1. Thus, in the COCA corpus, these words never appeared in immediately adjacent positions. Furthermore, all final critical pairs were chosen such that there was no overlap of constituents, and thus critical pairs, between Experiment 1 and Experiment 2.

*Filler words.* To limit relations between critical pairs and the surrounding context, filler words in positions 2 and 5 were selected carefully. Specifically, the word in position 2 was always a plural noun and the word in position 5 was always an adjective. Because plural nouns are almost never modifiers of subsequent nouns (Haskell, MacDonald, & Seidenberg, 2003), placing a plural noun in position 2 would discourage grouping with the noun in position 3. Similarly, because adjectives do not modify preceding words in English, placing an adjective in position 5 would discourage grouping with the noun in position 4.

Plural nouns were identified by selecting randomly from the list of head nouns that were not paired with noun modifiers. Adjectives were identified by searching the COCA corpus for the most frequent adjectives. Fillers in position 1 and 6 were selected from the same pool of fillers as in Experiment 1.

**Table S1.3. Noun compounds in Experiment 2.**

| Noun modifier | Head noun | Modifier freq. as modifier | Head freq. as head | Modifier prob. as modifier | Head prob. as head |
| --- | --- | --- | --- | --- | --- |
| parking | inquiry | 20493 | 10055 | .780 | .908 |
| refugee | nature | 6003 | 75014 | .742 | .907 |
| terrorist | size | 17211 | 59885 | .740 | .920 |
| vegetable | threat | 9368 | 36661 | .715 | .950 |
| twin | tendency | 10599 | 9908 | .658 | .990 |
| capitol | basis | 13799 | 38408 | .652 | .982 |
| executive | passage | 49888 | 17171 | .650 | .943 |
| breast | instruction | 20949 | 27100 | .649 | .904 |
| consumer | song | 24793 | 39894 | .648 | .905 |
| lemon | mode | 12974 | 12186 | .633 | .944 |
| voter | duty | 6619 | 18726 | .627 | .924 |
| cell | sauce | 36602 | 19727 | .607 | .906 |
| pine | sense | 10891 | 126006 | .604 | .926 |
| minority | luck | 22605 | 19258 | .600 | .935 |
| adult | weapon | 29561 | 15305 | .599 | .927 |
| peer | stability | 12917 | 14168 | .590 | .919 |
| deputy | room | 15719 | 201837 | .590 | .911 |
| wildlife | clothes | 15483 | 34679 | .582 | .941 |
| satellite | mouth | 11418 | 49360 | .580 | .956 |
| oak | analysis | 11565 | 68545 | .566 | .919 |
| silver | habit | 26190 | 9806 | .561 | .973 |
| graduate | failure | 21770 | 32096 | .553 | .940 |
| tobacco | floor | 14016 | 74019 | .548 | .911 |
| missile | portion | 10244 | 15394 | .539 | .970 |
| conservation | ingredient | 16342 | 3510 | .537 | .897 |
| ski | burden | 15324 | 14148 | .530 | .922 |
| resource | shirt | 16249 | 23452 | .529 | .903 |
| photo | discourse | 91757 | 11690 | .521 | .900 |
| welfare | trend | 22205 | 17992 | .516 | .926 |
| airline | supporter | 9237 | 4149 | .515 | .933 |
| steel | danger | 22740 | 23224 | .512 | .949 |
| security | narrator | 108849 | 15457 | .511 | .916 |
| storage | bunch | 15010 | 16753 | .500 | .950 |
| household | victory | 16365 | 29829 | .498 | .915 |
| fitness | window | 12360 | 60737 | .496 | .906 |
| toy | recipe | 8817 | 11604 | .495 | .899 |
| palm | meal | 15943 | 17285 | .490 | .913 |
| cookie | scandal | 5468 | 11515 | .490 | .942 |
| finance | evidence | 16414 | 90935 | .488 | .971 |
| intelligence | pair | 38172 | 28216 | .469 | .950 |
| gas | faith | 47749 | 37922 | .466 | .908 |
| utility | oven | 11200 | 14969 | .462 | .931 |
| literacy | throat | 12248 | 18530 | .458 | .941 |
| labor | nose | 42909 | 26570 | .457 | .928 |
| county | mechanism | 89934 | 9594 | .457 | .977 |
| bean | horizon | 6726 | 11670 | .454 | .906 |
| estate | mixture | 25435 | 19381 | .453 | .976 |
| fuel | accident | 27097 | 21489 | .447 | .914 |
| memorial | regulation | 15175 | 13945 | .446 | .926 |
| combat | column | 16676 | 15874 | .444 | .907 |
| rocket | priest | 7407 | 12296 | .438 | .896 |
| student | infection | 103135 | 13641 | .437 | .897 |
| credit | tongue | 45318 | 13920 | .435 | .910 |
| celebrity | knife | 10557 | 17673 | .433 | .917 |
| muscle | tragedy | 14771 | 12743 | .424 | .965 |
| volunteer | notion | 11793 | 22376 | .421 | .992 |
| cruise | lesson | 10381 | 20462 | .419 | .908 |
| holiday | crisis | 19274 | 41210 | .417 | .902 |
| radio | goal | 50739 | 47911 | .414 | .917 |
| jet | definition | 10789 | 18219 | .408 | .959 |
| designer | belief | 11727 | 20132 | .406 | .919 |
| fighter | wall | 6780 | 80108 | .405 | .902 |
| guest | lawsuit | 19947 | 11513 | .392 | .955 |
| defense | restriction | 74976 | 2392 | .389 | .902 |
| school | darkness | 338876 | 17447 | .380 | .973 |
| wage | gaze | 9851 | 14333 | .377 | .974 |
| forest | hell | 36117 | 36325 | .376 | .935 |
| energy | gesture | 83467 | 9098 | .375 | .964 |
| internet | tension | 43447 | 13699 | .374 | .947 |
| employment | pleasure | 17978 | 21616 | .373 | .901 |
| management | intention | 59322 | 9557 | .369 | .983 |
| championship | problem | 15948 | 156669 | .367 | .945 |
| climate | sky | 28118 | 41571 | .365 | .914 |
| police | origin | 133213 | 10558 | .364 | .920 |
| phone | creature | 91101 | 9861 | .363 | .926 |
| loan | illness | 12248 | 16369 | .362 | .917 |
| television | bag | 58599 | 36725 | .362 | .934 |
| stone | topic | 37492 | 15878 | .360 | .943 |
| navy | cheek | 18245 | 9591 | .358 | .960 |
| farm | religion | 30014 | 30249 | .350 | .909 |

**Table S1.4. Noun modifier and head noun SUBTLEXus frequencies in Experiment 2.**

| Noun modifier | Modifier SUBTLEXus log frequency | Head noun | Head SUBTLEXus log frequency |
| --- | --- | --- | --- |
| parking | 3.14 | inquiry | 2.25 |
| refugee | 2.00 | nature | 3.36 |
| terrorist | 2.79 | size | 3.37 |
| vegetable | 2.47 | threat | 3.03 |
| twin | 2.73 | tendency | 2.14 |
| capitol | 2.09 | basis | 2.79 |
| executive | 2.73 | passage | 2.59 |
| breast | 2.66 | instruction | 2.02 |
| consumer | 2.03 | song | 3.68 |
| lemon | 2.79 | mode | 2.42 |
| voter | 1.62 | duty | 3.42 |
| cell | 3.44 | sauce | 2.90 |
| pine | 2.50 | sense | 3.83 |
| minority | 2.06 | luck | 3.89 |
| adult | 2.86 | weapon | 3.38 |
| peer | 1.90 | stability | 1.96 |
| deputy | 2.90 | room | 4.35 |
| wildlife | 2.00 | clothes | 3.71 |
| satellite | 2.90 | mouth | 3.73 |
| oak | 2.46 | analysis | 2.75 |
| silver | 3.21 | habit | 2.87 |
| graduate | 2.80 | failure | 3.01 |
| tobacco | 2.55 | floor | 3.71 |
| missile | 2.83 | portion | 2.35 |
| conservation | 1.52 | ingredient | 2.13 |
| ski | 2.62 | burden | 2.70 |
| resource | 1.92 | shirt | 3.37 |
| photo | 3.07 | discourse | 1.32 |
| welfare | 2.61 | trend | 2.03 |
| airline | 2.52 | supporter | 1.58 |
| steel | 2.97 | danger | 3.35 |
| security | 3.68 | narrator | 2.72 |
| storage | 2.66 | bunch | 3.48 |
| household | 2.55 | victory | 3.04 |
| fitness | 1.95 | window | 3.64 |
| toy | 2.93 | recipe | 2.59 |
| palm | 2.83 | meal | 3.17 |
| cookie | 2.93 | scandal | 2.62 |
| finance | 2.44 | evidence | 3.64 |
| intelligence | 2.99 | pair | 3.28 |
| gas | 3.54 | faith | 3.37 |
| utility | 2.06 | oven | 2.66 |
| literacy | 1.42 | throat | 3.26 |
| labor | 2.79 | nose | 3.55 |
| county | 3.24 | mechanism | 2.28 |
| bean | 2.54 | horizon | 2.36 |
| estate | 3.03 | mixture | 2.10 |
| fuel | 2.94 | accident | 3.62 |
| memorial | 2.56 | regulation | 2.10 |
| combat | 2.85 | column | 2.75 |
| rocket | 2.78 | priest | 3.13 |
| student | 3.34 | infection | 2.65 |
| credit | 3.37 | tongue | 3.20 |
| celebrity | 2.57 | knife | 3.38 |
| muscle | 2.84 | tragedy | 2.86 |
| volunteer | 2.68 | notion | 2.53 |
| cruise | 2.80 | lesson | 3.22 |
| holiday | 3.04 | crisis | 2.93 |
| radio | 3.60 | goal | 2.93 |
| jet | 2.86 | definition | 2.47 |
| designer | 2.43 | belief | 2.59 |
| fighter | 2.81 | wall | 3.56 |
| guest | 3.31 | lawsuit | 2.49 |
| defense | 3.22 | restriction | 1.43 |
| school | 4.23 | darkness | 2.95 |
| wage | 2.20 | gaze | 2.11 |
| forest | 2.98 | hell | 4.38 |
| energy | 3.23 | gesture | 2.58 |
| internet | 2.53 | tension | 2.64 |
| employment | 2.44 | pleasure | 3.61 |
| management | 2.77 | intention | 2.75 |
| championship | 2.70 | problem | 4.23 |
| climate | 2.26 | sky | 3.36 |
| police | 4.08 | origin | 2.36 |
| phone | 4.14 | creature | 3.04 |
| loan | 3.01 | illness | 2.57 |
| television | 3.24 | bag | 3.68 |
| stone | 3.32 | topic | 2.43 |
| navy | 3.12 | cheek | 2.56 |
| farm | 3.19 | religion | 2.85 |

**S2. Model specification**

**S2.1. Model specification of Experiment 1.**

Recall of critical pairs (words in positions 3 and 4) were analyzed using generalized linear mixed effects regression (logistic) in which recall (1 = correct, 0 = incorrect) was regressed on consistency condition (-0.5 = inconsistent, 0.5 = consistent), position (-0.5 = 3, 0.5 = 4), and the interaction between consistency and position. A model with maximal random effects (a by-participant random intercept, a by-participant random slope for position, a by-participant random slope for consistency of the compound, a by-participant random slope for interaction between position and consistency of the compound, a by-item random intercept, and a by-item random slope for consistency of the compound) was attempted to be fit using the bobyqa optimization function. Steps at reducing model complexity followed guidelines of Brauer and Curtin (2017). A simplified model excluding the highest order by-participant random slope (by-participant random slope for the interaction between position and consistency of the compound) was chosen to avoid singularity of fit and to achieve convergence. Note that the exclusion of the highest order random effect inflates Type I error. The final model schematic follows, with intercepts denoted by “1” and random effects in parentheses: Accuracy ~ 1 + Consistency + Position + Consistency:Position + (1 + Consistency + Position|Participant) + (1 + Consistency|Word)

**S2.2. Model specification of Experiment 2.**

Recall of critical pairs (words in positions 3 and 4) were again analyzed using a generalized linear mixed effects regression (logistic) in which recall (1 = correct, 0 – incorrect) was regressed on consistency condition (-0.5 = inconsistent, 0.5 = consistent), position (-0.5 = 3, 0.5 = 4), ART score (centered), the interaction between consistency and position, and the interaction between consistency and ART score. The model was fit using the bobyqa optimization function. Again, a maximal random effects structure was attempted to be fit, though a simplified model needed to be used to achieve convergence and avoid singular fit errors. Steps at reducing model complexity followed guidelines of Brauer and Curtin (2017). The final model employed a by-participant random intercept, a by-participant random slope for consistency of the compound, a by-participant random slope for position, a by-item random intercept, a by-item random slope for consistency of the compound, and a by-item random slope for ART score. Note, higher interaction slopes were not included in the final model, which inflates Type I error. The final model schematic follows, with intercepts denoted by “1” and random effects in parentheses: Accuracy ~ 1 + Consistency + Position + ART + Consistency:Position + Consistency:ART + (1 + Consistency + Position|Participant) + (1 + Consistency + ART|Word)

**S2.3 Model specification of Experiment 3.**

As per the pre-registered analysis plan, old ratings (1 = Old, 0 = New) in the compound recognition task were analyzed using a generalized linear mixed effects regression (logistic) with fixed effects for oldness of the pair order (-0.5 = new; 0.5 = old), consistency of the compound at test (-0.5 = inconsistent; 0.5 = consistent), participant ART score (centered), the interaction of oldness of the pair order with ART score, and the interaction of the consistency of the pair at test with ART score. By-item and by-participant random intercepts were included, and the final model reported here included a by-participant random slope for oldness of the pair order, a by-participant random slope for consistency of the pair at test, a by-item random slope for oldness of the pair order, a by-item random slope for consistency of the pair at test, and a by-item random slope for ART score. The final reported model used the bobyqa optimization function. Note this model excludes higher order random slopes for interactions, which were excluded to achieve model convergence. Steps to achieve model convergence followed Brauer & Curtin (2017). The final model schematic follows, with intercepts denoted by “1” and random effects in parentheses: Rating ~ 1 + Oldness + Consistency + ART + Oldness:ART + Consistency:ART + (1 + Oldness + Consistency|Participant) + (1 + Oldness + Consistency + ART|Pair)

An additional model predicting old ratings was also fit including participants’ ratings of the meaningfulness of studied noun compounds as a covariate. This model included all of the same fixed effects as the previously reported model but with the additional covariate. The reported model included a by-participant random intercept, a by-participant random slope for the oldness of the compound at time of test, a by-participant random slope for the consistency of the compound at time of test, a by-item random intercept, a by-item random slope for the oldness of the compound at time of test, a by-item random slope for the consistency of the compound at time of test, and a by-item random slope for ART score. The final reported model used the bobyqa optimization function. The final model schematic follows, with intercepts denoted by “1” and random effects in parentheses: Rating ~ 1 + Oldness + Consistency + ART + Oldness:ART + Consistency:ART + Meaningfulness + (1 + Oldness + Consistency|Participant) + (1 + Oldness + Consistency + ART|Pair)

**References**

Brauer, M., & Curtin, J. J. (2018). Linear mixed-effects models and the analysis of nonindependent data: A unified framework to analyze categorical and continuous independent variables that vary within-subjects and/or within-items. *Psychological Methods*, *23*(3), 389. [https://doi.org/10.1037/met0000159](https://psycnet.apa.org/doi/10.1037/met0000159)
